# ESR statement on good scientific practice and publishing

**DOI:** 10.1007/s13244-012-0174-z

**Published:** 2012-05-23

**Authors:** European Society of Radiology (ESR)

**Affiliations:** Neutorgasse 9/2, 1010 Vienna, Austria

## Introduction

The European Society of Radiology (ESR) advocates best practice in the research process and requires all authors who submit their material to its peer-reviewed scientific publications (*European Radiology*, *Insights into Imaging*, *EPOS*, *EUROAD*, *ECR abstracts*) to comply with the highest ethical and scientific standards, as well as to its editorial policy of promoting and protecting the original authors’ scientific work.

## Research ethics

In biomedical research, it is of the utmost importance to comply with ethical principles and avoid any form of unethical experimentation in human or animal studies, adhere to ethical guidelines, seek ethics committee approval, obtain adequate subject consent and protect the privacy of the participants.

The fundamental document in the field of ethics in biomedical research that has influenced the formulation of international, regional and national legislation and codes of conduct is the Declaration of Helsinki [1], issued by the World Medical Association in 1964 [2]. This Declaration has been developed as a statement of ethical principles for medical research involving human subjects, including research on identifiable human material and data [1]. As well as following this statement of basic values of ethical biomedical research, investigators should follow all applicable rules and regulations, including national laws and regulatory guidelines. The Council for International Organizations of Medical Sciences (CIOMS), established jointly by WHO and UNESCO as an international, non-governmental organisation, has issued its International Ethical Guidelines for Biomedical Research Involving Human Subjects to serve the interests of the international biomedical community and help countries in defining national policies on the ethics of biomedical research involving human subjects [2]. Another basic source of reference is the Medical Ethics Manual, published by the World Medical Association [3].

One of the most important basic requirements in research involving patients or healthy test subjects is informed consent. This implies good communication, providing clear explanations of the procedures, treatments, risks, burdens and potential benefits. The approval of an ethics committee must be obtained; this committee should review the comprehensibility of the written communication, the soundness of the methodology, and assess the potential risks and burdens of the procedures in view of the anticipated benefits for the participants or the group they represent. Researchers should also make sure that the sample size is sufficiently high to provide statistically meaningful results and seek the early help of a statistician whenever appropriate. It would be unethical to subject patients (or animals) to risk and discomfort if a too-small sample size does not allow significant scientific conclusions [4]. The European Institute for Biomedical Imaging Research has compiled a document on best practice in biomedical imaging, which provides a valuable resource of basic premises and principles of ethical research [5].

Animal studies must follow the FELASA guidelines (Federation of European Laboratory Animal Science Associations) for animal use and for the training of persons involved in experimental animal care and use [6]. Before starting research involving animal experimentation, investigators must consider alternatives to painful procedures, minimise animal suffering, design a sound methodology and make sure that the knowledge to be gained is sufficiently important to justify the study. Authors can also refer to the guidelines issued by the National Institutes of Health [7, 8].

## Patient privacy

Patient privacy in medical research is a basic right of all study participants, and investigators must ensure that sensitive information about a person is thoroughly protected against unintentional leaks. Obviously, articles must not contain identifiable data of any sort within the text, tables and images. In addition, general principles and regulations on how to handle personal data to guarantee confidentiality should be adhered to. At the European level, this is regulated by the Directive 95/46/EC on the protection of personal data, which was implemented in 1995 by the European Commission [9]. The Directive sets strict limits on the collection and use of personal data, and sets out some of the highest standards of data protection in the world [10]. The principles defined in this Directive also apply to the transfer of data to countries outside the European Union, which is only permitted if the country ensures an adequate level of protection or the person controlling the data can guarantee that the recipient will comply with the data protection rules. Another useful reference for the planning of research with regard to the protection of patient data is the Personal Information in Medical Research guide provided by the UK Medical Research Council [11].

## Originality of submitted material

All manuscripts submitted to ESR scientific publications should be original work, created by the indicated author or author group. Gift authorship is strictly discouraged [12] and it is the authors’ sole responsibility to ensure that authorship criteria are fully met. If no part or specific task of a study can be attributed to a particular person, then this individual is not meeting the requirements for authorship credit.

In general, submission of a manuscript to one of ESR’s scientific publications strictly implies that the submitted work: (1) has not been published before, (2) is not under consideration for publication anywhere else and (3) has been approved for publication by all co-authors, if any, as well as by the responsible authorities, tacitly or explicitly, at the institute where the work has been carried out. If authors are planning to use their own previously published material to an extent that exceeds fair use, they must obtain permission from the copyright holder.

The International Committee of Medical Journal Editors (ICMJE) provides practical guidance to assist researchers in the preparation of manuscripts: The principles enshrined in Uniform Requirements for Manuscripts Submitted to Biomedical Journals [13] are incorporated into ESR’s publishing policies. Furthermore, ESR is also committed to the principles laid down by the Committee on Publication Ethics (COPE), such as the Code of Conduct and Best Practice Guidelines for Journal Editors [14]. COPE’s guidelines cover ethical, editorial and publishing issues as well as practical considerations regarding manuscript preparation.

Authors wishing to include images or text passages that have already been published elsewhere must obtain permission from the copyright owner(s) and include evidence that such permission has been granted when submitting their papers. Any material received without such evidence will be assumed to originate from the authors. Paraphrased material must be cited by source. If authors use information word for word, it should be in quotation marks with appropriate bibliographical citation. The accuracy and completeness of references in any submitted material is the sole responsibility of the authors.

Finally, even if information that is freely distributed in the public domain is used (e.g. taken from websites, etc.), the authors are profiting from the material and must still, therefore, receive permission. Occurrences of plagiarism are grounds for rejecting manuscripts.

## Conflict of interest declaration

It is the policy of ESR to ensure balance, independence, objectivity and scientific rigor in its publications. All authors are expected to disclose to the readers any real or apparent conflict(s) of interest that may have a direct bearing on the subject matter of the article. This pertains to relationships (remunerated or not) with pharmaceutical companies, biomedical device manufacturers or other corporations whose products or services may be related to the subject matter of the article. In particular, authors must acknowledge those companies who have supported the Department(s) where the work was carried out or may have sponsored the study in any way.

The intent of the policy is not to prevent authors who may have a conflict of interest from publishing their work. It is merely intended that any potential conflict should be identified openly so that the readers may form their own judgements about the article with the full disclosure of the facts. It is for the readers to determine whether the authors’ outside interest may reflect a possible bias in either the exposition or the conclusions presented.

In addition, it is now considered good practice for each published article to be countersigned by a ‘guarantor’ for the entire study. This may be the tenured senior author but it could be the Head of Department, Research Lead or other tenured staff member who is deemed to take overall responsibility for all aspects of the study (ethics, consent, data handling and storage and all other aspects of Good Research Practice). ESR uses this policy for original articles published in its journals [15].

## Scientific misconduct

Possible scientific misconduct includes: redundant publications (submitting the same article to different journals/media concurrently), data fabrication/falsification (intentional alteration of research processes and results as well as image manipulation), ‘salami slicing’ (dividing the same study/patient group into two or more articles and submitting them as different studies), overlapping publications (submitting a similar manuscript to different journals with different readerships), gift authorship (co-authorship awarded to a person with no or little involvement in the research process) and undisclosed conflicts of interest. It is appreciated that such some of these events may occur unintentionally or may sometimes arise out of naivety.

Copying ideas, writings, etc. from another and passing them off as one’s own is called plagiarism. Plagiarism can take many forms and vary in degree, but is considered as intellectual dishonesty.

As defined by the Committee on Publication Ethics (COPE) ‘Plagiarism ranges from the unreferenced use of others’ published and unpublished ideas, including research grant applications to submission under “new” authorship of a complete paper, sometimes in a different language. It may occur at any stage of planning, research, writing, or publication: it applies to print and electronic versions’ [16].

Accidental or unintentional plagiarism may occur when authors are largely unaware of citation and documentation rules guiding scientific practice. Inexperience with research methods, ignorance of citation rules and sloppiness are among the most common causes for this type of plagiarism.

## Detection of plagiarism and redundant publication

Reviewers for ESR’s scientific publications are chosen because they have intimate knowledge of the area of science in question and are usually fully conversant with the recent literature in their respective areas of expertise. Thus, the misappropriation of source material is often brought to light thanks to highly alert reviewers.

In addition, ESR is currently employing plagiarism-detection software to robustly detect highly and suspiciously similar written text. In instances where verbatim (word-for-word) plagiarism or redundant publication is suspected, the editors will carry out further investigation, with the help of editorial staff, expert reviewers and original copyright holders. If an initial suspicion is reliably confirmed, appropriate action will be taken, as recommended by COPE [14].

The first and obvious sanction in case of scientific misconduct, including plagiarism, is that the paper in question is not accepted by the journal or online publication. In other cases, some journals suggest that the (group of) author(s) are not entitled to submit another article for a certain period (e.g. a year or more).

It is recommended that authors always notify the editor of previous articles (exhibiting a high degree of similarity to the subject matter, theory, data or methodology used) on similar topics. Furthermore, the discussion of any article should clearly point out exactly how the current article builds on previous work and that it confirms/refutes previous published statements. If the editor, as a result of the author’s failure to disclose previous publication, is unaware of an apparent duplicate publication at the time of going to press, the journal reserves the right to issue a notice of duplicate publication and retract the paper as soon as redundancy is exposed. Similarly, other forms of serious scientific misconduct may lead to formal retraction of the paper.

Depending on the severity of the scientific misconduct, the editors may decide to impose sanctions against authors who have transgressed; this involves a letter to the Head of Department or University Dean, informing them about the malpractice and asking for an official explanatory statement [17].

## Management and execution of infringement

In cases of suspect behaviour, the corresponding authors and relevant superiors (such as the Head of Department, the Head of Institution or the Dean of the Faculty) will be informed.

Disciplinary steps can range, depending on the severity of the fraud, from publication of an apology or erratum, withdrawal of the article, to the refusal to consider more articles by that department or institution (e.g. for 6 months or up to 1 year). In the most serious cases of infringement or where the situation is debatable, the case could be referred to COPE (the Committee on Publication Ethics). Other Editors may be informed and the withdrawal of all articles by the respective author might even be considered.

## Conclusions

This paper has demonstrated some of the problems encountered by the Publications Department of the ESR and the way in which the Society reacts. Those issues related to publication in journals are in some ways the easiest to identify and deal with, as there is considerable written guidance on such issues. It is perhaps more difficult to spot duplication in case reports submitted to an online teaching file or abstracts to conference proceedings. However, the ESR is determined that exactly the same standards should be applied to all submissions to the Society. It is hoped that this article will act as a ‘wake up call’ to prospective authors and to Heads of Departments alike.
